# Ciliary Proteins: Filling the Gaps. Recent Advances in Deciphering the Protein Composition of Motile Ciliary Complexes

**DOI:** 10.3390/cells8070730

**Published:** 2019-07-17

**Authors:** Anna Osinka, Martyna Poprzeczko, Magdalena M. Zielinska, Hanna Fabczak, Ewa Joachimiak, Dorota Wloga

**Affiliations:** Laboratory of Cytoskeleton and Cilia Biology, Nencki Institute of Experimental Biology of Polish Academy of Sciences, 3 Pasteur Street, 02-093 Warsaw, Poland

**Keywords:** motile cilia, axoneme, dynein arms, MIPs, N-DRC, radial spokes

## Abstract

Cilia are highly evolutionarily conserved, microtubule-based cell protrusions present in eukaryotic organisms from protists to humans, with the exception of fungi and higher plants. Cilia can be broadly divided into non-motile sensory cilia, called primary cilia, and motile cilia, which are locomotory organelles. The skeleton (axoneme) of primary cilia is formed by nine outer doublet microtubules distributed on the cilium circumference. In contrast, the skeleton of motile cilia is more complex: in addition to outer doublets, it is composed of two central microtubules and several diverse multi-protein complexes that are distributed periodically along both types of microtubules. For many years, researchers have endeavored to fully characterize the protein composition of ciliary macro-complexes and the molecular basis of signal transduction between these complexes. Genetic and biochemical analyses have suggested that several hundreds of proteins could be involved in the assembly and function of motile cilia. Within the last several years, the combined efforts of researchers using cryo-electron tomography, genetic and biochemical approaches, and diverse model organisms have significantly advanced our knowledge of the ciliary structure and protein composition. Here, we summarize the recent progress in the identification of the subunits of ciliary complexes, their precise intraciliary localization determined by cryo-electron tomography data, and the role of newly identified proteins in cilia.

## 1. Introduction

**Figure 1:**
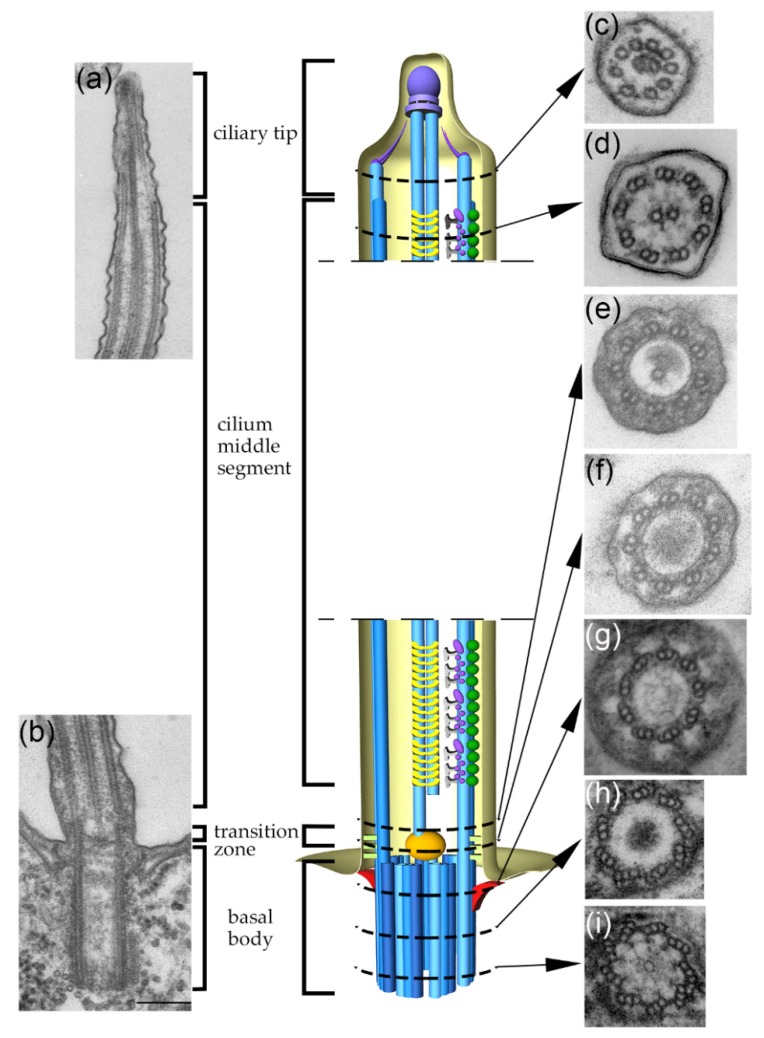
A schematic representation of the regions of the basal body and cilium. The basal body (microtubules in blue) is docked to the cell membrane through distal appendages called transition fibers (in red). The axonemal microtubules in the region of the transition zone (the most proximal part of the cilium) are connected to the ciliary membrane through Y-links (in light green). The most distal part of the cilium, called the ciliary tip, can structurally vary in cilia assembled by different cell types. Here, partial 10-protofilament microtubules (called B-tubules) of the outer doublets terminate earlier than 13-protofilament A-tubules. The plus ends of the ciliary microtubules are capped with the structure called the ciliary cap (in violet). The transmission electron microscopy (TEM) sections of a ciliate *Tetrahymena thermophila*. On the left are longitudinal TEM sections of (**a**) the ciliary tip, (**b**) the basal body, transition zone and part of the cilium. On the right are cross TEM sections of (**c**) the ciliary tip with central microtubules and peripheral singlets, (**d**) the main ciliary shaft, (**e**) region in which one of the central microtubules originates, (**f**) the transition zone, (**g**) the distal part of the basal body, (**h**) the middle part of the basal body, and (**i**) the proximal part of the basal body.

Motile cilia and homologous structures, flagella, are microtubule-based external cell protrusions assembled in eukaryotic organisms from evolutionarily distant lineages. Ciliary outer doublet microtubules are a continuation of two out of three microtubules of the basal body, a structure that anchors cilium to the cell body by so-called rootlets and docks to the cell membrane through transition fibers (Figure 1). The most proximal and distal regions of the cilium are called the transition zone and the ciliary tip, respectively, and they have unique ultrastructural organizations. Within the transition zone, the ciliary outer doublet microtubules are connected to the ciliary membrane through Y-shaped complexes (Y-links). The transition zone containing Y-links and the distal part of the basal body containing transition fibers form a ciliary gate that separates the cilium from the rest of the cell [1,2]. The microtubules terminate in the ciliary tip, and their plus ends are capped with a structure called a ciliary cap [3].

The activity of motile cilia is crucial for various motility-related biological processes, ranging from single-cell movements (e.g., sperm cells and some unicellular organisms) to the circulation or shift of fluids and particles along the surfaces of ciliated epithelial cells that line some human internal tracts. 

**Figure 2:**
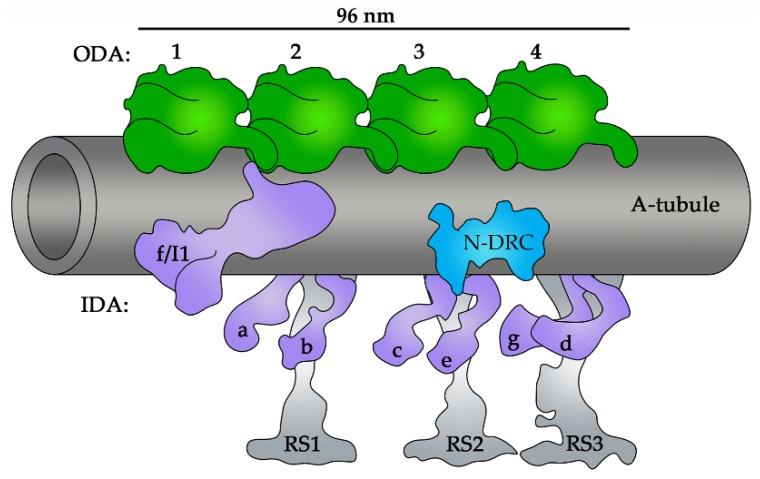
A schematic representation of the organization of macro-complexes within the 96 nm axonemal unit: ODAs (outer dynein arms, in green), IDAs (inner dynein arms, in violet), RSs (radial spokes, in grey), and the N-DRC (nexin–dynein regulatory complex, in blue) [4].

As previously mentioned, the skeleton of motile cilia is composed of uniquely arranged microtubules: nine peripheral microtubule doublets that are extensions of the basal body microtubules and two central microtubules. Microtubules serve as docking sites for periodically distributed protein complexes that are specific to either outer doublets or central microtubules. The outer doublet complexes form a specific pattern of 96 nm axonemal repeat units, which are distributed along the entire cilium length, except for a region of the ciliary tip. Each unit contains: (i) four identical outer dynein arms (ODAs) that, depending on the species, are either two- or three-headed, (ii) seven inner dynein arms (IDAs)—one two-headed (IDA f/I1) and six single-headed (IDAs a–e and g), which differ in their protein composition and function, (iii) three radial spokes (RSs), which functionally bridge the central apparatus and dynein arms, and (iv) a single nexin–dynein regulatory complex (N-DRC) that is postulated to be the main regulator of the complexes of an axonemal unit (Figure 2). The central pair complexes, which are called projections, are distributed either every 16 or 32 nm [4,5].

Large outer doublet complexes and central apparatus projections can be observed using classical transmission electron microscopy, and their general structure and protein composition are mostly well characterized. Recent studies of motile cilia using cryo-electron microscopy and 3D reconstruction (cryo-electron tomography, cryo-ET) have revealed new ultrastructural details of known macro-complexes and the existence of small complexes and linkers that connect different axonemal structures in the 96 nm axonemal unit. Not surprisingly, the protein composition and function of these recently discovered minor structures are mostly unknown. 

A full understanding of the molecular basis of ciliary beating at a small (axonemal unit) and large scales (whole cilium) requires the identification of most, if not all, of the proteins involved in this process. Genetic and proteomic studies on various organisms have concluded that several hundreds of proteins could be involved in the assembly and function of motile cilia [6,7,8]. However, to date, the precise intraciliary localization and function of a significant number of putative ciliary proteins are either unknown or poorly characterized. Recent years have seen significant progress in deciphering the protein composition of minor ciliary complexes and filling the “gaps” in the transduction of signals that control ciliary beating. This review briefly summarizes recent advances in the understanding of motile cilia ultrastructure and protein composition. This progress is the result of the joint efforts of researchers using genetic, biochemical, and cryo-ET approaches. 

## 2. Central Apparatus

The central apparatus is composed of (i) two 13-protofilament microtubules, C1 and C2, which are connected by (ii) a bridge-like structure, and (iii) several projections docked onto the C1 (C1a–C1f) and C2 (C2a–C2e) microtubules. Opposite to the outer doublet microtubules, two central microtubules originate in the proximal part of the cilium above the transition zone and extend to the ciliary tip. Central apparatus projections differ in size, architecture, and protein composition. In consequence, the central apparatus is asymmetric, both structurally and likely biochemically [9,10,11]. Presently, it is poorly understood how such asymmetry translates into ciliary beating and whether all projections can transiently interact with radial spokes.

Although the general arrangement of the central apparatus ultrastructure seems to be evolutionarily conserved, some details differ among species [5], and it is possible that these differences extend to their protein composition [11]. Initial comparative proteomic analyses of wild-type and central pair-less *Chlamydomonas* flagella revealed that the central apparatus is composed of at least 25 proteins, including α- and β-tubulins [12]. Some of these proteins do not have obvious orthologs in other organisms. A very recent study led to the identification of an additional 44 proteins that are likely subunits of the central apparatus in *Chlamydomonas.* However, only 13 of them have orthologs in humans [11]. Interestingly, mutations of some orthologous components of projections cause different changes in *Chlamydomonas* flagella and mammalian cilia [13].

In flagella assembled by *Chlamydomonas* and sea urchin sperm cells, the connecting bridge and most of the projections repeat every 16 nm. Exceptions are C1 projections c–f, which repeat every 32 nm [5]. *Chlamydomonas* PF20 (paralyzed flagella 20), a WD40-repeat-containing protein, an ortholog of mammalian SPAG16 (sperm-associated antigen 16), is proposed to be a component of the bridge-like structure. Interestingly, while one (30%) or, more frequently, two (70%) central microtubules are missing in the *Chlamydomonas pf20* mutant [14], both central microtubules are present in the sperm of SPAG16 mutant mice [15] and the flagella of the *Trypanosoma* PF20 knockdown mutant [16]. It is possible that the differences in central apparatus stability are due to the presence of additional structures in the flagella assembled by *Trypanosoma* (paraflagellar rod [17]) and mice sperm cells (dense outer fibers and fibrous sheath [18]). Thus, the lack of the central apparatus in green algae could be a secondary effect caused by the disruption of unstable microtubules during flagellar beating. 

In *Chlamydomonas*, C1a, one of the largest projections, is composed of at least six proteins: (i) PF6, an ortholog of mammalian SPAG17, (ii) calmodulin, and several as-yet uncharacterized proteins: (iii) FAP114 (flagella-associated protein 114) and (iv) FAP119, which have limited and high homology with mammalian CCDC189 (coiled-coil domain-containing protein 189), respectively, (v) FAP227, and (vi) LRR (leucine-rich repeat)-containing FAP101 [19,20,21]. The *Chlamydomonas pf6* immotile mutant lacks only the C1a projection [19], while tracheal cilia assembled in Spag17 knockout mice lack the C1a projection or the entire C1 microtubule [22]. The C1b projection in *Chlamydomonas* is composed of at least five subunits: (i) a large, adenylate kinase domain-containing protein, CPC1 (central pair-associated complex 1 [23]), an ortholog of mammalian SPEF2 (sperm flagellar protein 2 [24]), and several as-yet uncharacterized proteins: (ii) FAP69, an armadillo-repeat containing protein, (iii) enolase, (iv) HSP70, and (v) FAP42, a *Chlamydomonas*-specific 260 kDa protein that contains domains homologous to the guanylate and adenylate kinase domains [25,26]. The *Chlamydomonas cpc1* mutant lacks the C1b projection and often lacks C2b, which is likely stabilized by C1b [23,25]. Surprisingly, flagella assembled by the *Chlamydomonas cpc1* mutant are motile and beat with a normal waveform and a frequency that is reduced only by half [23,25]. Thus, at least in *Chlamydomonas*, the C1a projection seems to be more important than C1b in the generation of ciliary beating. 

The C1d projection is composed of the proteins FAP46, FAP54, FAP74, FAP297/WDR93 (WD-repeat containing protein 93), and FAP221/PCDP1 (primary ciliary dyskinesia protein 1) [27,28]. FAP46 and FAP74 are likely positioned at the base of the C1d projection [28]. *Chlamydomonas* cells with knocked down FAP74 or knocked out FAP46 have flagella that lack the C1d projection and the sheath between C1d and C1b [27,28] (please compare with the position of C1f [5]). Both *Chlamydomonas* mutants lacking C1d are mostly immotile or tumble in place; however, in some cells, flagella can still beat but with reduced frequency and a delay in the next effective stroke initiation [27,28]. 

Significantly less is known about the composition of C2 projections. Hydin is the only known component of the C2b projection, and knocking it down in green algae leads to the absence of C2b and a part of the neighboring C2c, and nearly complete paralysis of the assembled short flagella. In some flagella assembled by *hydin* mutants, one of the central microtubules (5–12%) or even the entire central apparatus (3-5%) is missing [29]. Similar defects (the absence of C2b and a part of the neighboring C2c) were observed in tracheal and ependymal cilia in *hydin* mutant mice [30]. Slowly beating or paralyzed flagella without C2c and C2b projections and the sheath material between them (please compare with the position of C2d [5]) were observed in *Chlamydomonas* cells with knocked down kinesin-like protein (KLP1), another component of the central apparatus [31,32].

The protein composition of the projections C1c, C1e, and C1f, as well as C2a, C2d, and C2e, remains unknown, although new candidate proteins were recently identified [11]. Furthermore, the precise localization of several proteins known to form the central apparatus needs to be determined. The list includes PF16/SPAG6, an armadillo repeat-containing protein required for C1 microtubule stability [33,34]; SPEF1/CLAMP (calponin-homology and microtubule-associated protein), which is likely a ciliary microtubule-associated protein that appears to be indispensable for central apparatus assembly or stability [35]; FAP174, which is homologous to MYCBP (C-myc binding protein); and putative AKAP240 (A-kinase anchoring protein 240), which is likely associated with the C2 microtubule [36], and serine–threonine protein kinase 36 (STK36) [37,38]. Interestingly, the entire central apparatus is missing in *Chlamydomonas* (pf15 [39], pf19, [40]) and *Tetrahymena* katanin mutants [41]. However, the significance of microtubule severing in central microtubules assembly remains an intriguing puzzle. 

While the absence of the entire central apparatus causes complete ciliary or flagellar paralysis, the outcome of the deficiency of a single projection or part of one can vary from total immotility to only slightly aberrant ciliary beating, depending upon the affected projection. Thus, it is probable that each projection of the central apparatus has its own (at least partly) specific function. 

## 3. Outer Doublets and Microtubule Inner Proteins

**Figure 3:**
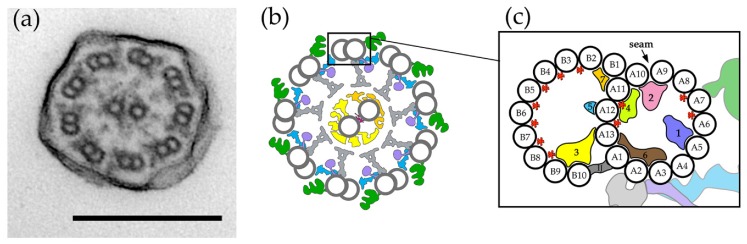
Organization of motile cilia ultrastructure: (**a**) transmission electron microscopy (TEM) cross-section of a *Tetrahymena* cilium, scale = 200 nm; (**b**) a schematic representation of the organization of axonemal macro-complexes: ODAs (outer dynein arms, in green), IDAs (inner dynein arms, in violet), RSs (radial spokes, in grey), the N-DRC (nexin–dynein regulatory complex, in blue), and the central apparatus (two microtubules, with their projections in yellow and dark yellow, and the bridge connecting the central microtubules in red); (**c**) schematic representation of the outer doublet showing protofilament numbering (A1–A13: protofilaments of the A-tubule, B1–B10: protofilaments of the B-tubule), position of the globular MIPs (microtubule inner proteins, 1–7), and the position of the outer doublet macro-complexes (colored as in (b)); the positions of the filamentous MIPs are marked by red stars. (**c**) Modified from [5,42,43,44,51].

The outer doublet microtubules (the so-called A- and B-tubules) are a continuation of two out of three basal body microtubules. In contrast to cytoplasmic microtubules and microtubules of the central apparatus, microtubules that form an outer doublet are permanently connected and thus have unusual architecture and stability. The A-tubule is a classical, 13-protofilament (A1–A13), cylinder-like structure, while the partial horseshoe-like B-tubule is composed of only ten protofilaments, B1–B10 [42]. The B-tubule is stably attached to the wall of the A-tubule at protofilament A1 (inner junction) and A10 (outer junctions) (Figure 3). Here, we follow the most frequent scheme used to number protofilaments (A1 and B10 protofilaments are at the inner junction, B1 and A10 are at the outer junction, and protofilaments are numbered anticlockwise; please note that the numbering is not correlated with the A-tubule lattice arrangement, as the seam of the A-tubule is positioned between protofilaments A9 and A10 [43,44]). 

The outer and inner junctions are structurally different. At the outer junction, the B1 protofilament is likely directly attached to the A-tubule wall at the A10 and A11 protofilaments [42,44]. The inner junction is formed by a non-tubulin ladder-like structure, previously described as non-tubulin protofilament B11, which connects A1 and B10 [42,45]. 

It was revealed that two evolutionarily conserved proteins form the inner junction in *Chlamydomonas:* FAP20/GTL3/C16orf80/BUG22 (basal body proteins with upregulated gene 22) [46] and PACRG (Parkin co-regulated gene) [47]. Both proteins are arranged alternately along the entire length of the flagellum with a periodicity of 8 nm for each protein [46,47,48]. Deletion of FAP20 or PACRG results in a less stable connection between A- and B-tubules and the lack of every other inner junction density (corresponding to either FAP20 or PACRG), while the entire inner junction structure is missing from flagella assembled by the double *fap20pacrg* mutant [46,47]. 

A growing number of studies have indicated that the luminal side of the wall of both A- and B-tubules serves as a docking site for numerous MIPs (microtubule inner proteins). As microscopic methods advance, the emerging picture becomes increasingly complex [4,42,43,44,45,49,50,51,52]. One of the recent analyses showed that there are two types of MIPs, i.e., globular (MIP1–7) and filamentous (fMIPs) [44]. Most globular MIPs are detected in the lumen of the A-tubule. Interestingly, some globular MIPs interact laterally with other MIPs and even protrude into the tubulin lattice [44]. 

The MIP1 density repeats every 8 nm and is formed in turns by larger MIP1a and smaller MIP1b densities, each repeating every 16 nm. In *Chlamydomonas,* the arch-like MIP1a spans protofilaments A5–A7 [42] or A5–A6 [43], while, in *Tetrahymena*, MIP1a is a smaller structure that is only visible at A5 [43]. MIP1b is attached exclusively to A5 [42,43,44,45,51]. 

MIP2 was initially described as a density that repeats every 16 nm along protofilaments A9 and A10 and is formed by two structures—MIP2a and MIP2b, arranged in the pattern MIP2a–MIP2a–MIP2b in a 48 nm unit [42,45]. One of the densities described previously as MIP2a is now named MIP2c [44,51]. Close to the MIP2 density is the MIP4 density (A10–A13) [42,43,44,45], which is composed of up to five substructures in *Tetrahymena* (MIP4a–4e) per 48 nm repeat [51]. The MIP6 density repeats every 8–16 nm and spans protofilaments A1–A3 [43,44]. Further analyses have revealed that MIP6 likely corresponds to four substructures, MIP6a–d [51].

MIP3, MIP5, and MIP7 are globular densities detected in the lumen of the B-tubule [42,43,44,45,51]. The organization of MIP3 resembles that of MIP1. The MIP3 density repeats every 8 nm along the B9–B10 protofilaments and is formed in turns by the larger arch-like MIP3a and the smaller MIP3b protein, each repeating every 16 nm [42,45]. The MIP5 structure binds every 16 nm to protofilament A12 [43], and the MIP7 density has been detected along A11 and B1 [44,51], corresponding to the structure described earlier as a laminar sheet [43].

In addition to globular MIPs, Ichikawa and co-authors [44] described filamentous MIPs (fMIPs), which are positioned in the grooves between protofilaments and, most likely, have a 48 nm periodicity. Four fMIPs were identified in the A-tubule (A6–A7, A7–A8, A11–A12, and A12–A13) and seven in the B-tubule (B2–B3, B3–B4, B4–B5, B5–B6, B6–B7, B7–B8, and B8–B9).

Despite advances in the localization of MIP densities, little is known about the identity of the proteins that constitute these structures. A few proteins, including tektin, Rib43a (ribbon protein 43a), and Rib72, are strongly attached to the protofilaments that are shared between the A- and B-tubule (A10–A13 and A1), thus increasing their stability [53]. Such localization corresponds to the position of some MIP densities. Rib72/EFHC1 (EF-hand domain-containing protein 1) is an evolutionarily conserved protein with three DM10 domains, which play a role in protein localization; in most orthologs, a C-terminal EF-hand domain is included [54]. *Tetrahymena* has two orthologs of Rib72: Rib72A/Bbc73 (basal body centriole protein 73) and Rib72B/Bbc60 [55]). Deletion of Rib72A results in the lack of MIPs 1a, 4b, 4d, 6b, and 6c and a reduction in MIP4e, while axonemes isolated from the *RIB72B-KO* mutant do not have structures corresponding to the MIP1b, 4e, 6a, and 6d densities. Accordingly, the double *RIB72A/RIB72B* mutant lacks the entire MIP1, MIP4, and MIP6 densities [51]. Thus, Rib72 is required for the assembly of these structures. From the estimated masses of the MIP1, MIP4, and MIP6 densities, the authors concluded that Rib72 is unlikely the sole component of these densities; instead, Rib72 could be a part of these structures [51]. 

Interestingly, in *Chlamydomonas*, another EF-hand motif-containing protein, FAP85, was suggested to form the A-tubule density. FAP85 localizes at the inner wall of the A-tubule with a periodicity of 48/50 nm [56]. However, FAP85 is encoded only by the genome of *Chlamydomonas* and related organisms. 

Deletion of the evolutionarily conserved proteins FAP45/CCDC19/NESG1 (nasopharyngeal epithelium-specific gene 1) and FAP52/WDR16 in *Chlamydomonas* causes the disappearance of the B-tubule-specific MIP densities MIP3c and MIP3a, respectively. In the double *fap45fap52* mutant, the stability of the B-tubule is compromised, and the density corresponding to IDA e is reduced [52]. Thus, it is possible that MIPs both stabilize the outer doublet microtubules and interact with some outer doublet complexes [51,52]. Such interactions are likely because some MIPs can protrude between protofilaments [44].

## 4. Ciliary Ruler

The characteristic, evolutionarily conserved arrangement of ciliary complexes in the 96 nm axonemal unit, together with the matching periodic distribution of the axonemal units along all nine outer doublets of the axoneme, is a fascinating biological phenomenon. Its origin was partly addressed by studies showing that two coiled-coil domain-containing ciliary proteins, FAP59/CCDC39 and FAP172/CCDC40, likely determine the length of the axonemal unit and a site of attachment for some ciliary macro-complexes [57]. 

FAP59 and FAP172 form a thin, approximately 96 nm long complex, and their ciliary localization is interdependent. The N-terminal ends of both proteins are positioned between the bases of RS1 (radial spoke 1) and RS2 of one axonemal unit, while the C-terminal ends of this linear complex reach between the bases of RS1 and RS2 of the subsequent axonemal unit [57]. The hypothesis that FAP59 and FAP172 may function as a “molecular ruler” is strengthened by the analyses of the *fap59fap172* double knockout, in which FAP59 and FAP172, each with duplicated corresponding protein fragments, were co-expressed. The co-expression of two mutant proteins results in the formation of a longer axonemal unit, with a length roughly proportional to the number of amino acids of the duplicated fragment [57]. Moreover, depending on the position of a duplicated fragment (N-terminal, middle, or C-terminal), additional structures are assembled in the lengthened axonemal units. Duplication of the N-terminal fragment causes the assembly of extra RS1, IDA a, and IDA f/I1 complexes, while an additional copy of the C-terminal part results in the presence of two N-DRCs, RS2, as well as IDA c and e in a single axonemal unit. In all cases, five instead of four ODAs are observed within a single lengthened unit [57]. Such observations have led to the hypothesis that the FAP59/FAP172 complex functions not only as a molecular ruler but also directly or indirectly regulates the recruitment of the subunits of other complexes to the axoneme [57]. This hypothesis is further supported by the observation that the lack of FAP59 or FAP172 in *Chlamydomonas* results in the assembly of flagella with axonemes containing fewer IDAs and N-DRCs, and both central microtubules and peripheral doublets are shifted from their natural positions [57]. Such structural alterations are also observed in the cilia of patients with PCD (primary ciliary dyskinesia) who have mutations in *CCDC39* and *CCDC40*, the human orthologs of FAP59 and FAP172, respectively [58,59,60]. 

ODAs are the only major axonemal outer doublet complexes whose 24 nm periodicity along the axoneme is not affected by the absence of FAP59 and FAP172. The mechanism of ODA pattern formation has not been fully elucidated. ODAs are attached to the A-tubules by the Outer Dynein Arm-Docking Complex (ODA-DC). In *Chlamydomonas*, the ODA-DC has an elliptical shape with a longer diameter of approximately 24 nm [61] and is composed of three subunits: two coiled-coil domains containing proteins DC1 (ODA3 [62,63]) and DC2 (ODA1 [64]) and a calmodulin-like protein, DC3 (ODA14 [65,66]). ODAs and ODA-DCs are assembled and transported to flagella independently of each other, and the binding of ODA-DCs to the A-tubule precedes the binding of ODAs [61,67]. Purified recombinant ODA-DC can dock onto in vitro polymerized microtubules; however, more than one complex can be bound per 24 nm microtubule fragment [61]. Similarly, up to four rows of the ODAs are observed if such microtubules are incubated with a fraction of axonemal complexes containing ODAs and the ODA-DC purified from *Chlamydomonas* flagella [68]. On the other hand, ODAs were shown to bind to microtubules even without ODA-DCs, and it was proposed that the 24 nm periodicity of the ODA attachment is due to intermolecular interactions and the physical size of ODAs. Thus, ODA-DCs could only stabilize docked ODAs [69]. However, it is not clear whether ODAs without ODA-DCs assemble into one or a few rows. Assembly into a few rows would suggest that some axonemal protein(s) restrict binding of ODA-DCs to a single row. 

It was postulated that the well evolutionarily conserved protein CCDC103, which strongly binds to the axoneme with a periodicity of 12 nm, could play a role in ODA positioning [70]. 

In humans, besides CCDC114 [71,72], an ortholog of ODA1/DC2, the docking (and/or targeting) of ODAs to cilia likely depends on the armadillo-repeat-containing protein ARMC4 [73] and the proteins CCDC151 [74] and TTC25 [75]. On the other hand, according to bioinformatics analyses, CCDC151 has some homology with *Chlamydomonas* ODA10. ODA10 and ODA5, which is orthologous to vertebrate LRRC56, are cytoplasmic proteins and interact with preassembled dynein arms before they are transported to flagella [76]. 

## 5. Outer and Inner Dynein Arms 

The protein composition, assembly, and function of dynein arms were recently reviewed [77]. Therefore, we only highlight some basic information and briefly summarize new discoveries. 

Dynein arms are large multi-protein complexes whose characteristic feature is the presence of the motor domain-containing dynein heavy chains. Depending on the analyzed species, ODAs contain two (e.g., vertebrates) or three (e.g., *Chlamydomonas*, *Tetrahymena*) dynein heavy chains (thus, two or three motor domains) and several other proteins called, depending on their molecular weight, an intermediate (IC) and light (LC) dynein chains. IDA f/I1 is the only two-headed IDA with its motor domains positioned relatively close to the surface of the A-tubule compared with other dynein arms [78]. In *Chlamydomonas*, besides two arm-specific dynein heavy chains, IDA f/I1 is composed of three intermediate chains (IC140/WRD63, IC138/WRD78, and IC97), a light intermediate chain (FAP120), and five light chains (LC7a, LC7b, LC8, Tctex1 (T-complex testis-specific protein), and Tctex2). The remaining six IDAs are single-headed with dynein heavy chains that are specific to each inner dynein arm. In addition to dynein heavy chains, they are composed of actin and either centrin (IDA b, e, g) or light chain p28 (IDA a, c, d) [77].

Several proteins required for the assembly of functional dynein arms act in the cytoplasm as co-chaperones (DNAAFs, dynein axonemal assembly factors) and participate in dynein arm preassembly. DNAAFs were discovered when it turned out that mutations in some non-axonemal proteins resulted in the lack of inner and outer dynein arms and cause PCD. Recently, it was shown that DNAAFs, together with chaperones and axonemal dyneins, form organelle-like structures called DynAPs (dynein axonemal particles) in multiciliated cells [79]. 

Because DNAAFs are not axonemal proteins, we only briefly list them here: DNAAF1/LRRC50, an ortholog of *Chlamydomonas* ODA7 [80,81,82]; DNAAF2/Ktu/PF13 [83]; DNAAF3/PF22 [84]; DNAAF4/DYX1C1/PF23 (dyslexia susceptibility 1 candidate 1) [85,86,87]; DNAAF5/HEATR2 (HEAT-repeat-containing protein 2) [88,89]; DNAAF6/PIH1D3 [90,91]; and CFAP300/C11orf70 [92,93]. Several other cytoplasmic proteins were also reported to play a role in dynein arm preassembly or transport, including LRRC6 [94,95,96], ZMYND10 [97,98,99,100], C21orf59/Kurly/FBB18 [101], WDR92 [102,103,104], and SPAG1 [105]. 

Transport of some ciliary components or preassembled complexes requires the presence of adaptor proteins. ODA16/WDR69 is a putative outer dynein arm adaptor that interacts with IFT46 [106,107,108].

Based on cryo-ET and biochemical analyses, it was recently proposed that CFAP70, a TPR (tetratricopeptide repeat) domain-containing protein, is tightly associated with axonemes, and its absence affects the beating of mouse ependymal cilia and *Chlamydomonas* flagella. Surprisingly, when FAP70 was expressed as an N-terminally BCCP-tagged fusion protein, an additional density corresponding to streptavidin staining was detected in two distinct positions: at the bases of the ODAs, suggesting its involvement in the regulation of ODA activity, and at the linker part of the N-DRC [109]. At the moment, whether CFAP70 is indeed a component of the N-DRC complex is unclear.

It is generally accepted that ciliary and flagellar beating is driven by the coordinated activity of the dynein arms. According to the widely accepted “switch-point” model, the active and inactive dyneins are positioned on opposite sides of the bent axoneme, and the status of the dyneins changes as the cilium bends in the opposite way [110]. Recent cryo-ET analyses of the flagella of rapidly frozen swimming sea urchin sperm cells showed that dyneins had different conformations corresponding to different states of their activity [111]. Subsequent mapping of dyneins in different conformations to the region of either principal or reverse bend, or the straight fragment of the axoneme inspired authors to form a compelling “switch-inhibition” model, in which dyneins are only locally inactivated during bending and are otherwise in an active state [111].

## 6. Nexin–Dynein Regulatory Complex

The Nexin–Dynein Regulatory Complex (N-DRC) is a large, approximately 50 nm long structure [112]. Initially, the N-DRC was characterized as two separate structures. Biochemical methods suggested the existence of a dynein arm regulator of unknown ciliary localization. At the same time, electron microscopy analyses revealed the presence of a bridge-like structure (called the nexin link [113]) between two neighboring outer doublets; the nexin link was believed to restrict the extent of the shift of the outer doublets generated by dynein arm activity [114,115]. It has since been proposed that the N-DRC not only plays a role in the conversion of doublet sliding into axonemal bending but also functions as a main hub that regulates and coordinates the activity of the macro-complexes in an axonemal unit [112]. 

Cryo-ET analyses of the N-DRC in *Chlamydomonas* flagella [112] and sea urchin sperm flagella [45] revealed that, although there are some differences in the N-DRC architecture, the N-DRC in both species is composed of two parts. A smaller, approximately 0.35 MDa fragment, called the base plate, attaches the complex to the surface of the A-tubule between the second and third radial spoke, starting at the inner junction between the A- and B-tubule and extending to protofilament A4 [112]. The remaining approximately 1–1.2 MDa part of the complex forms a large protrusion called a linker, which extends into the direction of the B-tubule of adjacent outer doublet [112]. Both the base plate and linker have minor protrusions that connect the N-DRC with different complexes in the 96 nm unit, including outer dynein arm linker 3 (OID linker 3a, [116,117]), an MIA complex (modifier of inner arms [118]), the head of the inner dynein arm IDA g, the tail of IDA e, and a base of RS2 [112]. 

In *Chlamydomonas*, an N-DRC complex is composed of at least eleven, mostly well evolutionarily conserved subunits, DRC1–11 [119,120,121]. Cryo-ET analyses of *Chlamydomonas* cells that either lacked specific DRCs or expressed tagged DRC proteins helped to determine the position of some of the DRC subunits within the N-DRC complex. The available data suggest that three coiled-coil domain-containing proteins (DRC1/CCDC164, DRC2/FAP250/CCDC65, and DRC4/PF2/GAS8) form the core of the N-DRC and function as a scaffold for the assembly of the remaining subunits. Their C-terminal fragments form a part of the N-DRC base plate and reach the doublet inner junction. DRC2 and DRC4 span the entire N-DRC, and their N-terminal ends extend to the very distal part of the N-DRC linker, called the distal lobe, while DRC1 terminates in the middle part of the linker [121,122,123]. Interestingly, the proper localization of DRC1 and DRC2 is interdependent, but in the absence of DRC1 or DRC2, some DRC4 still localizes in flagella. Moreover, in the *Chlamydomonas pf2* mutant, which lacks DRC4, the localization of DRC1 and DRC2 is preserved [119,120,122,123]. 

On the other hand, DRC4 is necessary for DRC3/FAP134/LRRC48, DRC5/FAP155/TCTE1 (Testis-Expressed Protein 1), DRC6/FAP169/FBXL13 (F-box and leucine-rich repeat protein 13), and DRC7/FAP50/CCDC135 docking, suggesting that DRC4 could function as a scaffold of these subunits [119,120]. Out of these four proteins, DRC3 forms the L1 protrusion of the N-DRC linker, with its C-terminal end positioned in the most distal part of L1, close to the surface of the microtubule, and the N-terminal end is located at the base plate–linker transition [122]. Loss of DRC3 does not affect the presence or localization of the other subunits [124], and the N-DRC of the *drc3* mutant only lacks densities corresponding to the L1 protrusion, a small part of the distal lobe, and a fragment connecting these two structures [124]. Immunoprecipitation data suggest that DRC3 directly or indirectly interacts with DRC4, DRC7, and DRC11/FAP82 [124]. 

Data concerning other N-DRC subunits: DRC8/FAP200/EFCAB2 (EF-Hand Calcium Binding Domain 2), DRC9/FAP122/IQCG (IQ Motif Containing G), and DRC10/FAP84/IQCD (IQ Motif Containing D) are still fragmentary. The C-terminal end of DRC5 is likely positioned in the middle part of the N-DRC linker, but the localization of the N-terminal end remains unknown [121].

A phenotype caused by the mutation in a particular N-DRC subunit depends on the subunit’s position in the N-DRC and thus the extent of the N-DRC damage. In humans, mutations of at least the core N-DRC subunits (DRC1, DRC2, and DRC4) result in PCD [125,126,127,128,129].

The mechanism by which the N-DRC regulates other complexes is an intriguing question. DRC3, positioned at the periphery of the complex, directly connects to the motor domain of IDA g and likely regulates its activity [124]. The N-terminal ends of DRC2 and DRC4 reach the distal lobe [121,122,123], a part of the N-DRC that interacts with the B-tubule of the adjacent outer doublet. The tubulin of the B-tubule is highly glutamylated [130,131]. Interestingly, the N-terminal end of DRC4 contains several lysines. It was proposed that these basic residues may interact electrostatically with the glutamic acid residues that are accumulated on the surface of the B-tubule [132]. The interactions between the N-DRC distal lobe and glutamylated surface of the B-tubule was also postulated by Alford and co-authors [133].

Some of the N-DRC subunits can be modified by phosphorylation [119]. Thus, it is possible that changes in the level of phosphorylation of DRC proteins (and responsible kinases and phosphatases) also affect the interactions between the N-DRC and other ciliary macro-complexes.

## 7. Radial Spokes 

Radial spokes (RSs) are T-shaped complexes that are visible as regularly distributed groups composed of three elements: RS1, RS2, and RS3. They are anchored perpendicularly to the A-tubule by the so-called stalk and extend the head toward the central apparatus. The stalk and head are connected by the so-called neck [134]. Each radial spoke of the triplet can be distinguished by the distance between them, and this is apparent even using TEM. The gap between RS1 and RS2 is 32 nm, and that between RS2 and RS3 is 24 nm, while the distance between RS3 and RS1 of the subsequent RS triplet is 40 nm [134]. 

In cilia assembled in the ciliate *Tetrahymena*, sea urchin, mouse, and human, all three RSs in the axonemal unit are of similar size, but their architecture is not identical, especially in the case of RS3. An extreme example of RS3 alteration is the short, knob-like structure in *Chlamydomonas* [45,135,136]. The morphological diversity of RSs may reflect the heterogeneity of the protein composition of the radial spokes and, in consequence, the functional diversity [136,137]. 

The proteins that form the radial spokes were mainly identified in *Chlamydomonas*. Comparative 2D gel analyses of axonemal proteins obtained from the wild-type and the RS1-RS2-spokeless *pf14* mutant revealed that radial spokes are composed of at least 23 polypeptides (radial spoke proteins, RSP1–23) [138,139,140]. Because both the wild-type and *pf14* mutant axonemes have the knob-like RS3 spoke, the identified 23 RSPs are likely subunits of RS1 and RS2 [45,135,136]. However, it is possible that in organisms assembling RS3 as a full-size structure, some of the RS1-RS2-specific proteins are also subunits of RS3. Further proteomic studies have revealed the RSP subsets that constitute the head (RSP1, 4, 6, 9, and 10), neck (RSP2, 16, and 23), and stalk (the remaining RSPs) regions of the radial spokes [134,135]. 

Not all RSPs identified in *Chlamydomonas* are evolutionarily conserved. RSP2, 5, 7, 8, 14, 15, and 17 either do not have obvious orthologs in humans or have only a small homologous protein fragment [140]. Thus, the question arises: in other organisms, does the composition of radial spokes partially differ from that in *Chlamydomonas*, or are there fewer proteins? 

Although the structures of radial spokes in the axonemal unit, especially those of RS1 and RS2, seem to be very similar, several studies have indicated that there are some differences in their protein composition. Depletion of RSP16/HSP40 (heat shock protein) in *Chlamydomonas* alters the structure of the neck and head of both RS1 and RS2, but to different extents [141]. In the ciliate *Tetrahymena*, knocking out FAP206, an evolutionarily conserved protein, results in the lack of RS2 (and rarely RS3 as well), while RS1 is unaffected. Cryo-ET analyses have shown that FAP206 forms a prong at the base of RS2 and is required for the stable docking of RS2 and the associated IDA c to the axoneme [142]. In contrast to FAP206, the knockout of either FAP61/C20orf26 or FAP251/WDR66 in *Tetrahymena* affects only the structure of RS3 and reduces the number of IDA d and g attached at the base of RS3 [137]. FAP61 is a part of the RS3 stem, while FAP251 forms an arch-like structure at the base of RS3 [137]. FAP61 and FAP251, together with FAP91/AAT-1 (AMY-1-associating protein expressed in testis 1 homolog) and calmodulin, were first described as subunits of the CSC complex (calmodulin and radial spoke-associated complex) in *Chlamydomonas* [143]. Flagella assembled by *Chlamydomonas* cells with a reduced level of FAP91 or FAP61 have a reduced number of RS3 or lack RS3, respectively. Moreover, the number of assembled RS2 was also reduced in both types of *Chlamydomonas* mutants [144,145]. Taken together, the data suggest that FAP61 is a subunit of the knob-like RS3 (likely together with the arch-forming FAP251; compare Figure 6C–D in [145] and Figure 3B in [137]). Thus, the second part of the CSC complex (Figure 6C–D in [145]), likely composed of FAP91, could form a prong at the base of RS2; both CSC parts are likely connected, although the nature of this connection is unclear (Figure 6C–D in [145]).

A number of *Chlamydomonas* RS proteins have specific domains or motifs: an AKAP (A-kinase anchoring protein) domain in RSP3, a MORN (Membrane Occupation and Recognition Nexus) domain in RSP1 and 10, a DPY-30 dimerization motif in RSP2, armadillo domain in RSP8, leucine-rich repeat in RSP15, DnaJ/HSP40 in RSP16, NDK (nucleoside diphosphate kinases) in RSP23/NME5 (non-metastatic cells 5), and the EF hand in RSP20 (calmodulin) [134,146,147,148]. The presence of certain domains and motifs can suggest the functions of these proteins. However, similar to the case of the central apparatus projections and IDAs, a question arises as to whether (i) all radial spokes participate equally in signal transduction from the central apparatus to the dynein arms, (ii) each spoke plays a specific role, or (iii) their roles partly overlap. The experimental data suggest that the last one can be correct. First, the tails of different inner dynein arms are docked at the base of different RSs: IDA a and b at the base of RS1, IDA c and e at the base of RS2, and IDA g and d at the base of RS3 [134,135,136]. Second, the docking sites of RS1 and RS2 are close to the two-headed IDA f/I1, while RS3 is positioned closer to the N-DRC [149]. Thus, each RS may transmit signals to the specific pair of the inner dynein arms and possibly regulate ciliary beating in a different manner. 

## 8. Small Complexes and Links

With the growing number of cryo-ET-based cilia analyses, it has become apparent that the organization of the 96 nm axonemal repeat unit is far more complex than assumed from classical TEM observations. It turns out that, besides long-known ciliary macro-complexes, each axonemal unit contains numerous tiny structures that vary in size and shape. With a few exceptions, their protein composition and role in cilia remain unknown.

**Figure 4:**
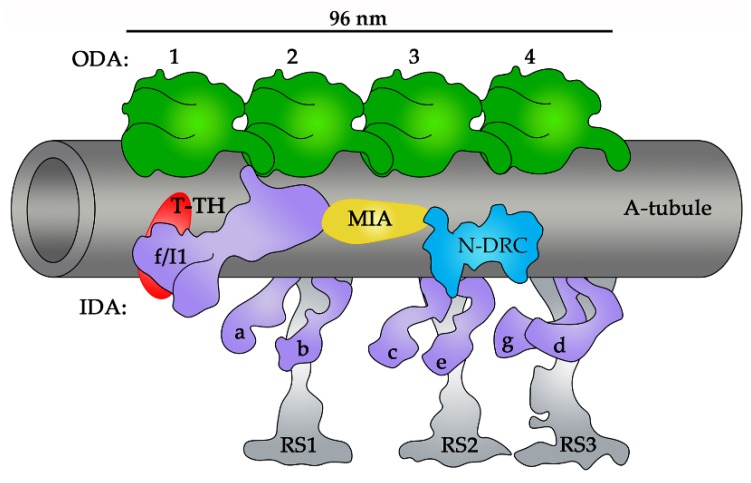
A schematic representation of the 96 nm axonemal unit with the position of the modifier of inner arms (MIA) complex (in yellow) and tether/tether head (T/TH) complex (in red) indicated. ODAs (outer dynein arms, in green), IDAs (inner dynein arms, in violet), RSs (radial spokes, in grey), N-DRC (nexin–dynein regulatory complex, in blue) [4,118,151,152,153].

Cryo-ET and biochemical analyses of the slow-motility *Chlamydomonas* mutants *mia1* and *mia2* led to the identification of an MIA (a modifier of inner arms) complex positioned between the N-DRC and intermediate/light chains-containing part of IDA f/I1 (Figure 4). The MIA complex likely directly interacts with IDA f/I1. The MIA complex is composed of two coiled-coil domain-containing proteins, FAP73/CCDC42 and FAP100/CCDC37 [118], and its absence increases the flexibility of IDA f/I1. Interestingly, the flagella of *mia1* and *mia2* mutants beat with reduced frequency, which suggests that MIA could also affect ODA activity [118], perhaps indirectly via IDA f/I1 [78,150,151].

Another small structure named tether/tether head complex (T/TH) was described as a density of unknown protein composition that is positioned in front of IDA f/I1 motor domains (Figure 4) and connects these motor domains to the surface of the A-tubule [151]. Subsequent studies of *Chlamydomonas* and *Tetrahymena* mutants revealed that T/TH extends to the base of RS3 and the tail of IDA d [152,153]. T/TH is composed of two proteins, FAP44/WDR52 and FAP43/WDR96, containing WD40-domains in their N-terminal fragment and coiled-coil domains in their C-terminal part [152,153,154]. 

In *Tetrahymena*, FAP43 and FAP44 localize along the entire cilium, and their localization is interdependent. The knockout of either protein causes the loss of the entire complex, which translates to an altered ciliary waveform and reduced beat amplitude [152,154]. 

Interestingly, in *Chlamydomonas*, FAP43 can be partly substituted by FAP244, a Chlorophyceae-specific protein [152,153]. In wild-type *Chlamydomonas* cells, FAP43 localizes along the flagellum, except for the short, proximal fragment that is most likely occupied by FAP244, while, in *fap244* mutants, FAP43 is present along the entire flagellum. On the other hand, in the *fap43* mutant, the level of FAP44 is diminished at the very distal end, suggesting that FAP244 is most likely unable to substitute for FAP43 in this region [153]. In the *fap43* and *fap244* single mutants, the tether complex seems to be mainly unaltered (86–90%), while the double *fap43fap244* mutant lacks the entire tether structure, similar to the *fap44* mutant [152,153]. Thus, in *Chlamydomonas*, besides the flagellar distal end, FAP43 and FAP244 are at least partly functionally redundant [152,153]. Cryo-ET analyses of *Chlamydomonas fap44* and *fap34fap244* mutants and the *Tetrahymena* FAP43 knockout mutant revealed that IDA f/I1 motor domains are shifted or occasionally missing, suggesting that T/TH likely restricts their movement and modulates IDA f/I1 activity [152,153].

## 9. From Basic Science to Human Health

Motile cilia serve as a locomotory organelle in unicellular organisms and sperm cells. In multicellular organisms, the coordinated beating of cilia generates a shift of the cells or fluids along the surface of the ciliated cells. In humans, motile cilia are assembled by the epithelial cells that line the upper respiratory tract, middle ear, brain ventricles, and, in females, the Fallopian tube. Their movement leads to the removal of mucus, inhaled particles, and bacteria from airways; the circulation of cerebrospinal fluid in brain ventricles; the transport of the oocytes in the Fallopian tube prior to fertilization; and sperm motility. The vortex-like movement of the nodal cilia, a specific type of motile cilia in the embryo node, facilitates the generation of the left–right asymmetry of the human body.

Defects in the assembly or function of motile cilia cause a multi-symptom, genetically heterogeneous disorder called primary ciliary dyskinesia (PCD), which manifests as chronic respiratory tract infections, infertility, and in about 50% of cases, laterality defects. This rare, inheritable disease affects 1 per 20,000 individuals [155]. To date, it has been shown that mutations in about 40 genes (accounting for less than 70% of all PCD incidences) lead to PCD [reviewed in 155]. Thus, the genetic background of a substantial portion of recognized PCD cases remains unknown. It is possible that the number of PCD-causative genes is much higher because individuals with the disease that experience only mild respiratory problems and lowered fertility might never be tested for PCD. Theoretically, a dysfunctional mutation in any gene encoding a structural or regulatory protein essential for motile cilia assembly or function can lead to PCD.

The identification of new proteins involved in the generation/regulation of ciliary beating and their functional analysis will lead not only to the broadening of the basic knowledge of ciliary motility but also help to establish new markers for PCD diagnosis. Interestingly, mutations in the recently described ciliary proteins CFAP43/FAP43, CFAP44/FAP44, and CFAP251/FAP251 cause MMAF (Multiple Morphological Abnormalities of the sperm Flagella), a syndrome that manifests as diverse sperm abnormalities and male infertility (reviewed in [156]). However, the molecular mechanisms behind the observed changes in sperm cells are unclear.

It is estimated that motile cilia are composed of several hundreds of proteins [6,7,8]. In addition to the structural components of the ciliary shaft, this number includes proteins such as those that build the transition zone [157,158,159] and ciliary tip [160,161,162]. Moreover, numerous proteins participate in intraciliary/intraflagellar transport (IFT), which is indispensable for cilia assembly and cilia length maintenance in the steady state [163,164,165], or perform enzymatic functions to locally regulate the amount of ATP or level of protein posttranslational modifications [25,26,166,167]. Without a doubt, advances in microscopy techniques and the combination of genetic, biochemical, bioinformatics, and cryo-ET approaches have led to substantial progress in deciphering the localization and function of ciliary proteins. Nevertheless, some aspects are only beginning to be understood, such as the composition of many minor ciliary structures, the nature of the reciprocal interactions between complexes, and the components of the regulatory pathways that locally and globally coordinate ciliary complexes and thus regulate ciliary motility.
